# 3D liver model-based surgical education improves preoperative decision-making and patient satisfaction—a randomized pilot trial

**DOI:** 10.1007/s00464-023-09915-w

**Published:** 2023-02-27

**Authors:** Esther Giehl-Brown, Sandra Dennler, Sebastián A. Garcia, Danilo Seppelt, Florian Oehme, Johannes Schweipert, Jürgen Weitz, Carina Riediger

**Affiliations:** 1 Department of Visceral, Thoracic and Vascular Surgery, University Hospital Carl Gustav Carus, Technische Universität Dresden, Dresden, Germany; 2 National Center for Tumor Diseases (NCT/UCC), Dresden, Germany; 3 German Cancer Research Center (DKFZ), Heidelberg, Germany; 4 Faculty of Medicine, University Hospital Carl Gustav Carus, Technische Universität Dresden, Dresden, Germany; 5 Helmholtz-Zentrum Dresden—Rossendorf (HZDR), Dresden, Germany; 6 Department of Radiology, University Hospital Carl Gustav Carus Dresden, Technische Universität Dresden, Dresden, Germany

**Keywords:** Three-dimensional visualization, Three-dimensional printing, Disease awareness, Validation research, Situational awareness, Preventive Healthcare

## Abstract

**Objective:**

Hepatobiliary surgery bares obstacles to informed consent for the patients due to its complexity and related risk of postoperative complications. 3D visualization of the liver has been proven to facilitate comprehension of the spatial relationship between anatomical structures and to assist in clinical decision-making. Our objective is to utilize individual 3D-printed liver models to enhance patient satisfaction with surgical education in hepatobiliary surgery.

**Design, setting:**

We conducted a prospective, randomized pilot study comparing 3D liver model-enhanced (3D-LiMo) surgical education against regular patient education during preoperative consultation at the department of Visceral, Thoracic and Vascular Surgery, University Hospital Carl Gustav Carus, Dresden, Germany.

**Participants:**

Of 97 screened patients, undergoing hepatobiliary surgery, 40 patients were enrolled from July 2020 to January 2022.

**Results:**

The study population (*n* = 40) was predominantly of male gender (62.5%) with a median age of 65.2 years and a high prevalence of preexisting diseases. Underlying disease, warranting hepatobiliary surgery, was malignancy in the majority of cases (97.5%). Patients in the 3D-LiMo group were more likely to feel very thoroughly educated and exhibited a higher level of satisfaction following surgical education than the control group (80 vs. 55%, n.s.; 90 vs. 65%, n.s.; respectively). Applying 3D models was also associated with enhanced understanding of the underlying disease with regard to amount (100% vs. 70%, *p* = 0.020) and location of liver masses (95 vs. 65%, *p* = 0.044). 3D-LiMo patients also demonstrated enhanced understanding of the surgical procedure (80 vs. 55%, n.s.), leading to better awareness for the occurrence of postoperative complications (88.9, vs. 68.4%, *p* = 0.052). Adverse event profiles were similar.

**Conclusion:**

In conclusion, individual 3D-printed liver models increase patient satisfaction with surgical education and facilitate patients’ understanding of the surgical procedure as well as awareness of postoperative complications. Therefore, the study protocol is feasible to apply to an adequately powered, multicenter, randomized clinical trial with minor modifications.

**Supplementary Information:**

The online version contains supplementary material available at 10.1007/s00464-023-09915-w.

Liver surgery is one of the most technically challenging procedures in the field of abdominal surgery due to the complex intrahepatic anatomy of the vasculature and biliary tree with frequent variations. In the last decades, advancements in perioperative patient care have increased the safety of liver resections [1, 2], directly increasing the number of patients suitable for surgery. Simultaneously, advancements in resection techniques have let to more complex surgical procedures. As a result, literacy of the proposed surgery as well as the underlying disease become even more relevant to allow for informed consent. Surgeons play an important role in this process, satisfying the informational need of patients while setting realistic expectations. Yet, opportunities to engage the patients during preoperative consultation are frequently missed [3–5].

3D visualization has gained broad applications in preoperative planning and intraoperative navigation particularly in hepatobiliary surgery [6, 7]. Routine two-dimensional computed tomography (CT) angiography provides a restricted apprehension of spatial relation of anatomical structures [8]. Therefore, intraoperative ultrasound remains an indispensable tool, but remains highly dependent on the surgeon’s experience [6]. 3D visualization of the liver addresses these hurdles [7]. With the development of 3D reconstruction software and 3D printers, patient-specific 3D liver models become more accessible. [9] 3D-printed liver models have already been applied to preoperative planning, surgical evaluation and intraoperative management of liver diseases, thereby changing clinical practice [7, 10–12]. Virtual or printed 3D liver models have proven to be more advantageous to surgeons in visualizing the spatial relationship between anatomical structures in comparison to two-dimensional images [13, 14]. A recent systematic review by Emile et al. showed positive effects of printed 3D models in surgery of colon cancer with liver metastases- not only in resection planning, but also in patient education [15]. Yang et al. used patient-specific, printed 3D liver model in parental education before liver surgery of children with hepatic tumors. Usage of 3D models improved literacy of the liver anatomy, disease characteristics and the proposed resection technique [16]. In general, sufficient preoperative counseling, that satisfies the patient’s informational curiosity, is benefiting post-operative recovery in surgical patients [17].

While the educational benefit of 3D visualization in the clinical setting for the operating surgeons- especially young surgeons has been explored previously [13, 18], studies on the potential benefit for patients in liver surgery are limited. Here, we explore the application of individual 3D liver models during preoperative consultation to reinforce engagement of the patient and thereby promote involvement in the decision-making process. We propose that individual 3D-printed liver models subsequently result in superior patient satisfaction with surgical consultation. Moreover, we suggest that facilitated understanding of the surgical procedure also influences the postoperative course through enhanced patient compliance.

## Material and methods

### Study design

**Fig. 1:**
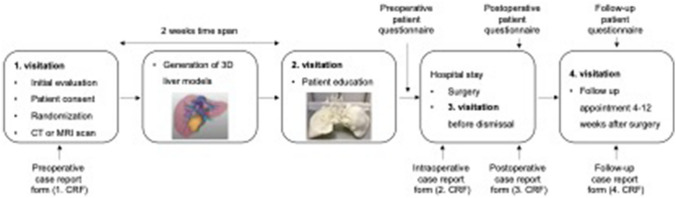
Flow diagram of the study protocol including time points of scheduled patient visitations, generation of the 3D liver models and data acquisition

This study was designed as a prospective, non-blinded, randomized controlled, monocentric pilot trial [19]. Patients were prospectively randomized into an intervention (3D-LiMo) group or control group using block randomization technique with blocks of 5 patients each. Randomization was performed by independent study nurses from the departments own center for clinical trials. Surgical education of patients, randomized into the control group, was performed using standard education sheets provided by Thieme (© 2022 Thieme Compliance GmbH) in addition to individual drawing by the surgeons and CT or MRI 2D images. Patients in the 3D-LiMo group received surgical education enhanced by patient-specific 3D-printed liver model. Figure 1 displays the time schedule of the study. Comprehension of the surgical procedure and satisfaction with patient education were anonymously inquired through questionnaires at different time points (Fig. 1). All questions were presented with 5 answering choices: very true, true, undecided, not true, absolutely not true. All aspects of the clinical trial were anonymously documented in the specific case report forms (CRFs) at different timepoints (Fig. 1).

### Patient population

97 patients, presenting in the outpatient clinic of the Surgery Department in the University Hospital Dresden for liver surgery between July 2020 and January 2022, were screened for inclusion. Inclusion criteria implied age above 18 years, indication for liver resection and preoperative CT imaging. In addition, a 2 weeks minimum time gap between enrollment and preoperative consultation was required for manufacturing of the 3D-printed models. A language barrier, lack of compliance or cognitive impairment were considered exclusion criteria.

### Virtual and printed 3D- liver models.

**Fig. 2:**
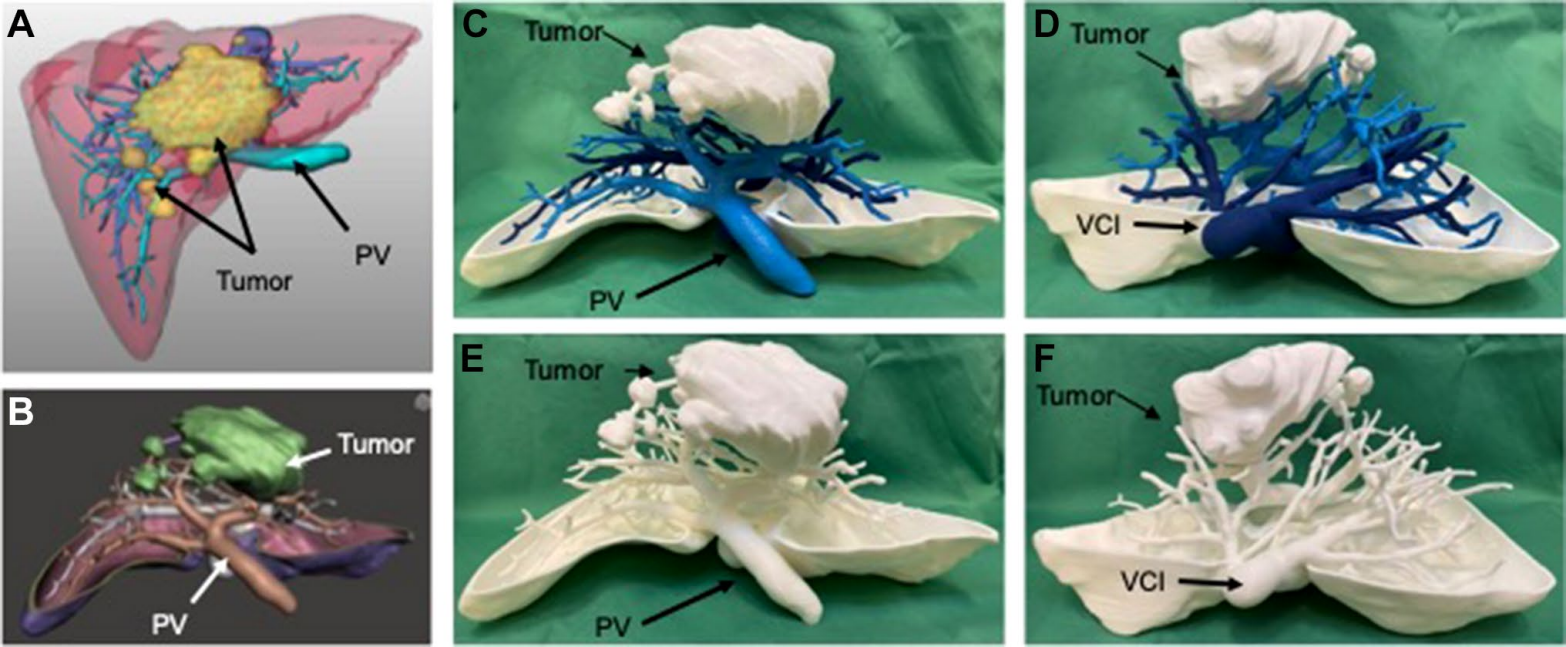
**A**–**B** Virtual 3D liver model provided by MeVis ©, displaying the intrahepatic portal veins (PV), hepatic veins branching in the inferior vena cava (VCI), as well as the liver tumor nodules; **C**–**D** Hand-colored printed 3D liver model displaying dividing portal and hepatic venous branches for demonstration purposes; **E**–**F** Personalized printed 3D liver model as used in the trial. *Star symbol* right liver lobe

High resolution CT images with a maximum thickness of 5 mm and intravenous contrast medium were needed to identify and characterize the intrahepatic vessels (portal vein and its branches, hepatic veins and their tributaries). 3D reconstruction was carried out by MeVis (MeVis Medical Solutions AG, Bremen, Germany) through its MeVis-Distant-Services (Fig. 2A). The STL files produced by MeVis containing the parenchyma, hepatic veins, portal vein and liver masses were modified using the Meshmixer (Autodesk, California, USA) program. The reconstruction of the hepatic parenchyma was transformed into a hollow object and about one third of its ventral side was sectioned. Once the editing was completed, it was exported as a single STL file. The Ultimaker Cura 4.7 Software (Ultimaker, Utrecht, Netherlands) was used to generate the G-CODE files. Models were printed on a scale of 1:1 using the Ultimaker S5 (Ultimaker, Utrecht, Netherlands) (Fig. 2B, D). Polylactic acid (PLA) tough white with a layer height of 0.2 mm was used for the liver model and Polyvinyl alcohol (PVA) for its support (Fig. 2C). 3D-printed parts were left overnight in a water bath to remove the PVA.

### Study endpoints

The primary endpoint of this study was patient satisfaction with surgical education. Secondary endpoints included understanding of the planned surgery, surgery length, conversion to open surgery, postoperative blood transfusions, postoperative morbidity and mortality, need of surgical revision, postoperative interventions, postoperative length of intensive care unit (ICU) stay and postoperative length of hospital stay.

### Statistics

The Kolmogorov–Smirnov test was used to assess distribution of investigated parameters. Categorical and quantitative variables were analyzed using Fisher’s exact test and unpaired t-test, respectively. Data was expressed as mean±standard deviation. Numeric variables were expressed as median with interquartile range (IQR). The values *P* < 0.05 were considered statistically significant. Statistical analysis was carried out using the software IBM SPSS 25 (SPSS Statistics V25, IBM Corporation, Armonk, New York).

### Ethical aspects and trial registration

The study was conducted according to the Declaration of Helsinki with waivers of informed consent of all patients. Ethical approval by local ethics committee was obtained before analysis (Number: BO-EK-168052020). The current study was registered at the German Clinical Trials Register (Deutsches Register Klinische Studien (DRKS)) with the number DRKS00022397.

## Results

### Patient’s characteristics

*N* = 40 patients were enrolled in this study and subsequently equally randomized into two study groups (*n* = 20 3D-LiMo group and *n* = 20 control group). The study population consists of 62.5% male and 37.5% female patients with a median age of 65.2 years. Patients showed a high prevalence of preexisting cardiovascular, gastrointestinal and endocrine diseases (65, 50 and 35%, respectively, Table 1). The majority of enrolled patients underwent any kind of previous surgeries (82.5%). 97.5% of enrolled patients suffered from malignant disease. Main underlying diagnosis and indication for surgery were colorectal liver metastases (52.5%) and hepatocellular carcinoma (22.5%). The comparison of the two groups revealed no significant difference in distribution of basic characteristics (Table 1).

**Table 1:** Basic patient characteristics

	Total *n* = 40	3D-LiMo *n* = 20	Control *n* = 20	*p*-value
Gender [*n* (%)]				0.74
Female	15 (37.5)	7 (35)	8 (40)	
Male	25 (62.5)	13 (65)	12 (60)	
Median age [years (range)]	65.2 (27–81)	67.55 (30–81)	62.85 (27–79)	0.18
BMI [mean (SD)]	28.7 (5.4)	27.8 (5.4)	29.6 (5.4)	0.29
Pre-existing disease [n (%)]				
Cardiovascular disease	26 (65)	14 (70)	12 (60)	0.51
Gastrointestinal disease	20 (50)	12 (60)	8 (40)	0.21
Endocrine disease	14 (35)	7 (35)	7 (35)	n.s
Neurologic /psychiatric disease	6 (15)	1 (5)	5 (25)	0.18
Liver cirrhosis [*n* (%)]	2 (5)	1 (5)	1 (5)	n.s
Liver steatosis [*n* (%)]	7 (17.5)	4 (20)	3 (15)	n.s
Regular alcohol consumption [*n* (%)]	14 (35)	4 (20)	10 (50)	0.11
Nicotine abuse [*n* (%)]	6 (15)	3 (15)	3 (15)	n.s
Medication [*n* (%)]				
ASS	5 (12.5)	4 (20)	1 (5)	0.34
New oral anticoagulants	2 (5)	1 (5)	1 (5)	n.s
Warfarin	2 (5)	2 (10)	0 (0)	0.49
Insulin	2 (5)	1 (5)	1 (5)	n.s
Previous surgery [*n* (%)]	33 (82.5)	19 (95)	14 (70)	0.09
Colorectal (CR) liver metastases	21 (52.5)	10 (50)	11 (55)	
Cholangiocarcinoma	3 (7.5)	1 (5)	3 (15)	
Hepatocellular carcinoma	11 (22.5)	7 (35)	4 (20)	
Liver metastasis other than CR	3 (7.5)	2 (10)	1 (5)	
Hepatolithiasis	1 (2.5)	–	1 (5)	

### Patients’ satisfaction with surgical education and perception of the 3D liver model.

Immediately after surgical and anesthesiological education, patient’s satisfaction was inquired via questionnaire. No significant differences were observed with satisfaction with overall medical care, medical care by surgeons or nurses, surgical preparations in general and anesthesiological preparations (Table 2). Nevertheless, 25% more patients of the 3D-LiMo group felt very well educated about the planned surgery in comparison to the control group. In addition, a higher percentage of patients felt very satisfied with medical care in the surgical outpatient clinical (90 vs. 65%, respectively) and with the surgeons (85 vs. 65%, respectively) in the 3D-LiMo group in comparison to the control group (Table 2). We also inquired about patients’ perception of the individual 3D-printed liver models. The majority of patients from the 3D-LiMo group claimed a better understanding of the proposed liver surgery (very true—70%, true—20%). Moreover, patients indicated an improved feeling and enhanced security about the proposed surgical procedure (very true—60%, true—30% vs. very true—65%, true—5%; respectively). Although the medical management of all patients was identical, patients of the 3D-LiMo group felt better taken care of due to the 3D liver model (very true—70%, true—30%). We inquired about the occurrence of anxiety and confusion in reaction to the 3D-printed liver models. All patients of the intervention group denied the occurrence of anxiety and confusion during preoperative education due to the usage of 3D models (Table 2).

**Table 2:** Preoperative patient questionnaire

	Total *n* = 40	3D-LiMo *n* = 20	Control *n* = 20	*p*-value
Satisfaction				
How thoroughly educated do you fell about the planned surgery? [*n* (%)]				
Very well	27 (67.5)	16 (80)	11 (55)	0.15
Well	11 (27.5)	4 (20)	7 (35)	
Undecided	2 (5)	–	2 (10)	
How satisfied are you with medical care in the surgical outpatient clinic overall?				
Very satisfied	31 (77,5)	18 (90)	13 (65)	0.16
Satisfied	8 (20)	2 (10)	6 (30)	
Undecided	1 (2,5)	–	1 (5)	
How satisfied are you with medical care by the surgeons in the outpatient clinic?				
Very satisfied	30 (75)	17 (85)	13 (65)	0.28
Satisfied	8 (20)	2 (10)	6 (30)	
Undecided	2 (5)	1 (5)	1 (5)	
How satisfied are you with medical care by the nurses in the outpatient clinic?				
Very satisfied	29 (72.5)	15 (75)	14 (70)	0.34
Satisfied	10 (25)	5 (25)	5 (25)	
Undecided	1 (2.5)	–	1 (5)	
How satisfied are you with surgical preparations in general?				
Very satisfied	29 (72.5)	15 (75)	14 (70)	0.60
Satisfied	10 (25)	5 (25)	5 (25)	
Undecided	1 (2.5)	–	1 (5)	
How satisfied are you with preparations for anesthesia in the outpatient clinic?				
Very satisfied	30 (75)	16 (80)	14 (70)	0.50
Satisfied	8 (20)	3 (15)	5 (25)	
Undecided	2 (5)	1 (5)	1 (5)	
Comprehension				
Patient correctly specified the surgery in her/his own words [*n* (%)]	37 (92.5)	20 (100)	17 (85)	0.20
Patient selected the correct type of surgery from a presented list [*n* (%)]	27 (67.5)	16 (80)	11 (55)	0.18
Patient stated the correct amount of masses to be removed [*n* (%)]	34 (85)	20 (100)	14 (70)	*0.020*
Patient located the masses in the correct liver lobe(s) [*n* (%)]	32 (80)	19 (95)	13 (65)	*0.044*
Patient correctly knew whether surgery will be performed open or laparoscopic [*n* (%)]	38 (95)	20 (100)	18 (90)	0.50
3D liver model				
Due to the 3D liver model, I have a better understanding of the surgical procedure				
Very true		14 (70)		
True		4 (20)		
Undecided		2 (10)		
Because of the 3D liver model, I have a better feeling about the planned surgery				
Very true		12 (60)		
True		6 (30)		
Undecided		2 (10)		
As a result of the 3D liver model, I feel more secure about the planned surgery				
Very true		13 (65)		
True		5 (5)		
Undecided		2 (10)		
Because of the 3D liver model, I feel better taken care of				
Very true		14 (70)		
True		6 (30)		
Due to the 3D liver model, I am more frightened of the surgery				
Not true		2 (10)		
Absolutely not true		18 (90)		
The 3D liver model confused me				
Not true		1 (5)		
Absolutely not true		19 (95)		

### Patient’s apprehension of the underlying disease and planned surgery

We evaluated patient’s understanding of the planned surgical procedure following surgical education. First, patients had to name the surgical procedure in their own words, we saw that most patients referred to unspecific terms (e.g. removal of mass) without clarifying mass count and location or type of resection (52.5%, data not shown). When asked to pick the correct type of liver surgery from a displayed list (atypical resection, anatomic resection, hemi-hepatectomy, associating liver partition and portal vein ligation for staged hepatectomy, left or right lobe), 80% of patients from the 3D-LiMo group and 55% of the control group correctly selected the surgical procedure (*p* = 0.176). Although significance was not reached due to small population size, application of the 3D model increased apprehension of the surgical procedure by 25%  (Table 2). Moreover, patients of the 3D-LiMo group were significantly more often capable of selecting the correct number of liver masses as well as their in comparison to the control group (*p* = 0.02 and 0.044 respectively) (Table 2).

### Surgical procedures and postoperative clinical course

The control and 3D-LiMo groups displayed no differences in the length of the operation, intraoperative blood loss and other characteristics of the surgical procedure. Also, none of the patients experienced adverse events during surgery (Table 3). During 11 out of 40 surgeries, a change of surgical concept occurred (5 and 6 times in the 3D-LiMo and control groups, respectively). Causes for change of concept included open conversion, impossibility of curative resection due to peritoneal carcinomatosis, small size of the potential liver remnant or underestimated number or size of malignant masses (e.g. additional colorectal liver metastases). Patients, who experienced a deviation of their surgical concept in comparison to the proposed procedure, were not excluded from the study. All cancer patients were offered psychosocial oncology support.

**Table 3:** Specification of surgical procedure, postoperative clinical course and postoperative patient questionnaire

	Total *n* = 40	3D-LiMo *n* = 20	Control *n* = 20	*p*-value
Specification of surgical procedure				
Surgical procedure [*n* (%)]				0.44
Atypical resection	12 (30)	7 (35)	5 (25)	
Anatomical resection	7 (17.5)	3 (15)	4 (20)	
Bisegmentectomy	5 (12.5)	–	5 (25)	
Right hemihepatectomy	5 (12.5)	3 (15)	2 (10)	
Left hemihepatectomy	2 (5)	2 (10)	–	
ALPPS	6 (15)	3 (15)	3 (15)	
Exploratory laparotomy	3 (7.5)	2 (10)	1 (5)	
Mean time of operation [mean (SD) in min]	230.7 (115.8)	249.8 (141.8)	211.5 (81.7)	0.30
Mean intraoperative blood loss [mean (SD) in ml]	711.0 (768.5)	683.2 (821.4)	737.5 (735.2)	0.87
Surgical approach [*n *(%)]				
Open	33 (82.5)	18 (90)	15 (75)	
Laparoscopic	3 (7.5)	–	3 (15)	
Open conversion	4 (10)	2 (10)	2 (10)	
Change of surgical concept [*n* (%)]	11 (27.5)	5 (25)	6 (30)	n.s
Count of Pringle maneuver [*n* (%)]				0.70
0	21 (52.5)	12 (60)	9 (45)	
1–3	17 (42.5)	8 (40)	9 (45)	
> 3	2 (5)	–	2 (10)	
Occurrence of intraoperative adverse events	–	–	–	
Postoperative clinical course				
Postoperative ultrasound [*n* (%)]	21 (27.5)	10 (50)	11 (55)	n.s
Postoperative computed tomography (CT) [*n* (%)]	20 (50)	12 (55)	9 (45)	n.s
Occurrence of postoperative ascites [n (%)]	12 (30)	7 (35)	5 (25)	n.s
Postoperative blood transfusions [mean (SD) in units]	1.75 (7.4)	3.1 (10.3)	0.4 (1.0)	0.25
Postoperative complication [*n* (%)]	19 (47.5)	9 (45)	10 (50)	0.5
Postoperative intervention [n (%)]	9 (22.5)	4 (20)	5 (25)	0.50
PTCD*	1	1	-	
Upper endoscopy, ERCP*	3	3	-	
Interventional angiography	3	2	1	
US- or CT-guided drainage*	5	1	4	
Portal vein embolization	2	1	1	
Occurrence of surgical revision [*n* (%)]	6 (15)	4 (20)	2 (10)	0.66
Postoperative mortality [*n* (%)]	2 (5)	2 (10)	–	0.24
Length of intensive care unit stay [mean (SD) in days]	2.7 (8.2)	4.3 (11.4)	1.1 (1.6)	0.21
Length of hospital stay [mean (SD) in days]	17.6 (13.7)	20.1 (16.1)	15.0 (10.5)	0.43
Patient satisfaction	*n* = 38	*n* = 18	*n* = 20	
Preoperative visitation by operating surgeon [*n* (%)]:	25 (65.8)	11 (61.1)	14 (70)	0.41
Postoperative visitation by operating surgeon [*n* (%)]:	31 (77.5)	16 (80)	15 (75)	0.66
Satisfaction with medical care at the ward? [*n* (%)]				
Very satisfied	20 (52.6)	9 (50)	11 (55)	0.76
Satisfied	15 (39.5)	7 (38.9)	8 (40)	
Undecided	2 (5.3)	1 (5.6)	1 (5)	
Dissatisfied	1 (2.6)	1 (5.6)	–	
Satisfaction with care by physicians at the ward? [*n* (%)]				
Very satisfied	28 (73.7)	13 (72.2)	15 (75)	0.56
Satisfied	8 (21.1)	4 (22.2)	4 (20)	
Undecided	1 (2.6)	–	1 (5)	
Dissatisfied	1 (2.6)	1 (5.6)	–	
Satisfaction with care by the nurses at your ward? [*n* (%)]				0.22
Very satisfied	25 (68.4)	11 (61.1)	15 (75)	
Satisfied	9 (23.7)	4 (22.2)	5 (25)	
Undecided	3 (7.9)	3 (16.7)	–	
Satisfaction with your hospital stay overall? [*n* (%)]				n.s.
Very satisfied	20 (52.6)	10 (55.6)	10 (50)	0.93
Satisfied	16 (42.1)	7 (38.9)	9 (45)	
Undecided	2 (5.3)	1 (5.6)	1 (5)	
Patients’ comprehension	*n* = 38	*n* = 18	*n* = 20	
Patient correctly stated whether the surgery was carried out as planned? [*n* (%)]	31 (83.3)	16 (88.9)	15 (78.9)	0.66
Patient correctly recalled the occurrence of postoperative complications? [*n* (%)]	30 (81.1)	17 (94.4)	13 (68.4)	*0.052*

*PTCD percutaneous transhepatic cholangiography, *ERCP* endoscopic retrograde cholangiopancreatography, *US* ultrasound, *CT* computed tomography

Complications in the clinical postoperative course occurred in both groups. Nevertheless, no significant differences in the number of postoperative complications between the two groups were observed (45% and 50% of the 3D-LiMo or control group, respectively (Table 3)). Complications included infected hematomas, bile leackage or biloma, wound healing deficits, lymphatic fistula, burst abdomen, COVID-19 infection, paralytic ileus, deep venous thrombosis, lung embolism, pleural effusions, postoperative bleeding, sepsis, liver failure and delirium. We also compared length of ICU and total hospital stay and were not able to detect distinctions (Table 3).

Postoperative patient satisfaction was assessed on the day of dismissal from the surgical ward. The majority of patients in the control and 3D-LiMo group were visited by the operating surgeon before (70% and 61.1%, respectively) and/ or afterwards (75 and 80%, respectively). Patients in the 3D-LiMo group were significantly more likely to correctly answer, whether postoperative complications have occurred, in comparison to the control group (94.4 vs. 68.4%, respectively, *p* = 0.52). No significant difference was detected when comparing satisfaction with medical care and hospital stay overall (Table 3).

### Follow-up evaluation 4 to 12 weeks after hospital discharge

No differences in the occasion of complications, recurrence of cancer or death was assessed (Supplementary table 1). First, we assessed that 95% of control population correctly recalled the event of complications in comparison to 67% of patients in the 3D-LiMo group. Second, we evaluated patient’s health awareness and satisfaction at the time of follow-up. 75% of control patients were very satisfied with surgical education in retrospect while only 61% of 3D-LiMo patients classified themselves of that kind. Nevertheless, the rest of 3D-LiMo patients (39%) was still satisfied (Supplementary table 1).

## Discussion

The present study analyzes whether a personalized 3D-printed liver model manifests an educational benefit to patients, encourages their involvement in the decision-making process and thereby enhances patient’s satisfaction. As surgical procedures of the liver have become more complex and are frequently associated with postoperative complications, we applied patient questionnaires to examine the level of surgical comprehension and level of satisfaction with surgical education when 3D liver models were applied in addition to preexisting informational sheets. Overall, patient satisfaction with preoperative preparations was very high in general (Table 2, 75 vs. 70% in the 3D-LiMo or control groups, respectively). Most patients were either very satisfied or satisfied with surgical (Table 2; totalized 95% in either study groups) and anesthesiological care (Table 2; totalized 95% in both groups). Although no significant differences were observed with satisfaction with overall medical care, surgical and anesthesiologic preparations; we observed higher percentages in the 3D-LiMo group in comparison to the control group. 80% of patients in the 3D-LiMo group felt very thoroughly educated about the planned surgical procedure in comparison to 55% in the control population (Table 2). This trend was also visible in higher satisfaction with the surgical outpatient clinic (90 vs. 65% in the 3D-LiMo and control groups, respectively; Table 2) and the surgeons (85 vs. 65% in the 3D-LiMo and control groups, respectively; Table 2), which might be indirectly linked to the printed liver model. Interestingly no difference in percentages was observed with satisfaction with the nurses and preparations in general. We did not observe a saving of time due to the application of the 3D models during preoperative consultation.

The current study revealed that surgical education applying 3D models was associated with enhanced disease understanding as 3D-LiMo patients were significantly more likely to correctly point out the number of liver masses (100 vs. 70% in the 3D-LiMo and control groups, respectively; *p* = 0.02; Table 2) as well as their location (95 vs. 65% in the 3D-LiMo and control groups, respectively; *p* = 0.044; Table 2). 3D-LiMo patients also demonstrated enhanced understanding of the surgical procedure as they were more likely to correctly select the surgical procedure from a displayed list (80 vs. 55% in the 3D-LiMo and control groups, respectively; Table 2). The need for interventions to improve preoperative decision-making and manage postoperative expectations has been addressed by previous studies [4, 5]. The application of visual and haptic 3D liver models could play a pivotal role in engaging patients in preoperative decision-making process and prepare them for expected and unexpected outcomes. When questioned about the postoperative clinical course, patients of the 3D-LiMo group revealed a significantly increased awareness for the occurrence of postoperative complications (94.4% vs. 68.4% in the 3D-LiMo and control groups, respectively; *p* = 0.052; Table 3). Although no significant differences in the postoperative occurrence of complications or post-operative recovery were detected, we do suggest that enhanced health literacy leads to increased patient compliance. The level of literacy and satisfaction of patient’s knowledge on their disease and the planned surgical procedure is positively correlated with the length of hospitalization and the incidence of postoperative complications in general surgery patients [17, 20]. To our knowledge, no clinical studies evaluating this influence on patients undergoing liver surgery exists yet. We suggest that the small study population is responsible for the lack of a difference in postoperative outcome in our study.

We were not able to detect an effect of the 3D model on patient satisfaction at the end of the hospital stay or at the follow-up appointment. We suggest that satisfaction levels at these time points are more dependent on the medical care at the surgical ward including nurses and physicians, occurrence of complications and the overall success of the surgery. The two study groups did not display a significant difference in morbidity, mortality, length of intensive care unit or total hospital stay.

The present study has limitations. First, the pilot trial design of the study leads to a small study population of 40 patients in total. This small number of enrolled patients lowered the likelihood of detecting significant differences. Nevertheless, this study serves as proof of principle for the application of 3D liver visualization during preoperative consultation and the justification of future clinical studies. Second, this study was conducted as a single center experience. Nevertheless, as we aimed to assess educational benefit and satisfaction in patients, we suggest that this limitation did not affect our results and conclusions. Third, the high requirements of the CT scan and time expenditure of generating the 3D-printed models individually for each patient resulted in low recruitment rates. Resource-intensive, patient-specific 3D models pose an additional expenditure to hospitals. Despite this direct increase in financial costs, we suggest that application of 3D models reduce overall costs of hospitalization eventually. Despite our focus on improvement of patient’s literacy of their disease and the planned surgical procedure, we see the advantage of 3D models in their diverse application. Individualized 3D anatomical models of the liver can improve pre-operative planning through enhanced visualization of the specific patient’s liver anatomy and facilitated comprehension of anatomic relation of tumors to the intrahepatic vasculature [21]. In addition to pre-operative planning, 3D visualization can be used for navigation during liver resection. In comparison to regular 2D visualization, application of 3D models positively impacts the operation itself by decreasing blood loss, operation time and the occurrence of postoperative complications [22, 23].

## Conclusion

In conclusion, individual 3D-printed liver models increase patient satisfaction with surgical education immediately following patient education. Besides this comforting value, liver models implicate an educational benefit for patients and reinforce patients’ engagement in the decision-making process. Patients of the 3D-LiMo group were significantly more likely to correctly state the number and location of liver masses. Enhanced understanding of the surgical procedure in the 3D-LiMo group transferred into a higher awareness of postoperative complications. As a result, the current study protocol is feasible to apply to an adequately powered, multicenter, randomized clinical trial with minor modifications. Based on our finding, we strongly recommend the application of 3D-printed models during patient education whenever available.

## Supplementary Information

Below is the link to the electronic supplementary material.

Supplementary file1 (JPG 11 KB) A PVA supported patient-specific printed 3D liver model.

Supplementary file2 (DOCX 305 KB)
