# Supplementary figures and images for: 3D liver model-based surgical education improves preoperative decision-making and patient satisfaction—a randomized pilot trial
Source: Surg Endosc. 2023 Feb 27;37(6):4545–54. doi: 10.1007/s00464-023-09915-w (PMC9970129; doi:10.1007/s00464-023-09915-w)

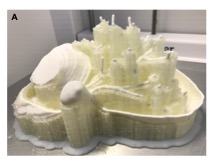

Supplement: Supplementary file 1 — Supplementary file1 (JPG 11 KB) A PVA supported patient-specific printed 3D liver model. [file 464_2023_9915_MOESM1_ESM.jpg]
