# Supplementary material for: 3D liver model-based surgical education improves preoperative decision-making and patient satisfaction—a randomized pilot trial
Source: Surg Endosc. 2023 Feb 27;37(6):4545–54. doi: 10.1007/s00464-023-09915-w (PMC9970129; doi:10.1007/s00464-023-09915-w)
Supplement: Supplementary file 2 [file 464_2023_9915_MOESM2_ESM.docx]

Supplementary table 1. Follow up and patient questionnaire 4-12 weeks following surgery.

	Total n=38	3D-LiMo n=18	Control n=20	p-value
**Outpatient clinical course**				
Occurrence of complications after dismissal [n (%)]	8 (21.1)	4 (22.2)	4 (20)	n.s.
Postoperative mortality [n (%)]	3 (7.9)	2 (11.1)	1 (5)	n.s.
Recurrence of cancer after curative resection [n (%)]	2 (5.3)	1 (5.5)	1 (5)	n.s.
**Patients' comprehension**				
Patient correctly recalled the occurrence of complications after dismissal [n (%)]	31 (81.5)	12 (66.7)	19 (95)	*0.038*
**Patients' self-awareness and satisfaction**				
How do you judge your general condition? [n (%)]				n.s.
- excellent	8 (21.1)	3 (16.7)	5 (25)	
- good	14 (36.8)	6 (33.3)	8 (40)	
- mediocre	10 (26.3)	7 (38.9)	3 (15)	
- poor	4 (10.5)	2 (11.1)	2 (10)	
- very poor	2 (5.3)	-	2 (10)	
How do you judge your general condition? [n (%)]				n.s.
- excellent	9 (23.7)	4 (22.2)	5 (25)	
- good	13 (34.2)	6 (33.3)	7 (35)	
- mediocre	10 (26.3)	6 (33.3)	4 (20)	
- poor	4 (10.5)	2 (11.1)	2 (10)	
- very poor	2 (5.3)	-	2 (10)	
How do you evaluate your mental health? [n (%)]				n.s.
- excellent	7 (18.4)	3 (16.7)	4 (20)	
- good	13 (34.2)	4 (22.2)	9 (45)	
- mediocre	10 (26.3)	7 (38.9)	3 (15)	
- poor	6 (15.8)	4 (22.2)	2 (10)	
- very poor	2 (5.3)	-	2 (19)	
Satisfaction with medical care overall? [n (%)]				n.s.
- very satisfied	21 (55.3)	11 (61.1)	10 (50)	
- satisfied	15 (39.5)	7 (38.9)	8 (40)	
- undecided	2 (5.3)	-	2 (19)	
Satisfaction with patient education in retrospectiv? [n (%)]				n.s.
- very satisfied	26 (68.4)	11 (61.1)	15 (75)	
- satisfied	11 (28.9)	7 (38.9)	4 (20)	
- undecided	1 (2.6)	-	1 (5)	
